# Antibodies Elicited by the *Shigella sonnei* GMMA Vaccine in Adults Trigger Complement-Mediated Serum Bactericidal Activity: Results From a Phase 1 Dose Escalation Trial Followed by a Booster Extension

**DOI:** 10.3389/fimmu.2021.671325

**Published:** 2021-05-04

**Authors:** Francesca Micoli, Omar Rossi, Valentino Conti, Odile Launay, Antonella Silvia Sciré, Maria Grazia Aruta, Usman Nasir Nakakana, Elisa Marchetti, Rino Rappuoli, Allan Saul, Laura B. Martin, Francesca Necchi, Audino Podda

**Affiliations:** ^1^ GSK Vaccines Institute for Global Health, Siena, Italy; ^2^ Faculté de Médecine Paris Descartes, Université de Paris, Paris, France; ^3^ Inserm CIC 1417, F-CRIN I-REIVAC, Paris, France

**Keywords:** *Shigella sonnei*, GMMA, serum bactericidal activity, dose escalation, booster response, 1790GAHB

## Abstract

*Shigella* is the second most deadly diarrheal disease among children under five years of age, after rotavirus, with high morbidity and mortality in developing countries. Currently, no vaccine is widely available, and the increasing levels of multidrug resistance make *Shigella* a high priority for vaccine development. The single-component candidate vaccine against *Shigella sonnei* (1790GAHB), developed using the GMMA technology, contains the O antigen (OAg) portion of lipopolysaccharide (LPS) as active moiety. The vaccine was well tolerated and immunogenic in early-phase clinical trials. In a phase 1 placebo-controlled dose escalation trial in France (NCT02017899), three doses of five different vaccine formulations (0.06/1, 0.3/5, 1.5/25, 3/50, 6/100 µg of OAg/protein) were administered to healthy adults. In the phase 1 extension trial (NCT03089879), conducted 2–3 years following the parent study, primed individuals who had undetectable antibody levels before the primary series received a 1790GAHB booster dose (1.5/25 µg OAg/protein). Controls were unprimed participants immunized with one 1790GAHB dose. The current analysis assessed the functionality of sera collected from both studies using a high-throughput luminescence-based serum bactericidal activity (SBA) assay optimized for testing human sera. Antibodies with complement-mediated bactericidal activity were detected in vaccinees but not in placebo recipients. SBA titers increased with OAg dose, with a persistent response up to six months after the primary vaccination with at least 1.5/25 µg of OAg/protein. The booster dose induced a strong increase of SBA titers in most primed participants. Correlation between SBA titers and anti-*S. sonnei* LPS serum immunoglobulin G levels was observed. Results suggest that GMMA is a promising OAg delivery system for the generation of functional antibody responses and persistent immunological memory.

## Introduction

Diarrheal diseases continue to be a major cause of death worldwide, with approximately 1.6 million fatalities estimated in 2017 (1). Although mortality rates from diarrheal diseases have decreased since 1990 (1), diarrhea morbidity remains high, particularly in low- and middle-income countries, lacking relevant microbiological diagnostics, water quality, and sanitation, and adequate health-care facilities and treatment interventions are not easily accessible (2, 3). Complications from repeated infections are especially common in malnourished children, with possible long-term consequences including stunting and intellectual deficit (4–6). *Shigella* is the second leading cause of diarrheal disease after rotavirus and is the main pathogen associated with diarrhea in children under five years of age in developing countries (7–9). *Shigella* has also been associated with diarrhea in adults, with increased disease incidence in the elderly (10). Moreover, it is a leading cause of diarrhea in travelers and military personnel (11–13).

The genus *Shigella* comprises four species (*S. flexneri*, *S. sonnei*, *S. dysenteriae*, and *S. boydii*), with more than 50 serotypes differentiated based on the structure of the somatic O antigen (OAg), the polysaccharide moiety of the lipopolysaccharide (LPS) anchored in the outer membrane of the bacteria (14). OAg is the component involved in many pathogen-host interactions and is a key antigen recognized by the immune system following natural infection (14). It has been shown that individuals infected with *Shigella* acquire natural immunity that prevents or reduces severity of recurrent infections caused by the same serotype (15–19). This protective immunity was associated with the level of LPS-specific serum immunoglobulin G (IgG) and immunoglobulin A (IgA) antibodies during several disease outbreaks (11, 19). Individuals with a high level of anti-LPS serum antibodies showed significantly reduced disease severity (20). Therefore, current vaccine development strategies against *Shigella* mostly target the serotype-specific OAg of the bacteria. Several vaccine candidates, developed using different techniques, are under investigation (21–25), but no vaccine is widely available. However, the morbidity of the disease coupled with the rise of antimicrobial resistance (26, 27) urges for the introduction of an effective vaccine.

*Shigella*-specific antibody response following both natural infection and vaccination has traditionally been evaluated using antigen-specific enzyme-linked immunosorbent assay (ELISA). This serological method relies on antibody binding to an immobilized antigen and does not provide information on the functionality of the antibody. Although protection against *Shigella* is mediated by multiple mechanisms that are not yet fully elucidated, antibodies against OAg can fix complement and kill *Shigella* (28, 29). Bactericidal antibody activity has been identified in adults from endemic regions who mounted immunity after natural exposure (30). Even if an immunological correlate of protection is not established for *Shigella*, antibodies with bactericidal activity are expected to be a relevant indicator of protective immunity facilitating the development of vaccines against *Shigella* (31–33). A recent study reported a strong association between *S. flexneri* 2a-specific serum bactericidal activity (SBA) titers in human adult volunteers and reduced clinical disease following challenge with wild-type bacteria (34), thus supporting the value of bactericidal antibodies to potentially predict vaccine efficacy.

1790GAHB is a single-component *S. sonnei* candidate vaccine, developed using the GMMA-technology (23, 35) as a delivery system for OAg. The vaccine has been demonstrated to induce anti-LPS serum IgG antibodies and to have an acceptable safety profile in phase 1 trials conducted in adults from France and the United Kingdom (36) and in a phase 2a trial in an adult population from Kenya, where *Shigella* is endemic (37). A booster dose of 1790GAHB administered in an extension trial 2–3 years following the primary vaccination was well tolerated and induced an anamnestic response in adult French participants (38).

**Figure 1:**
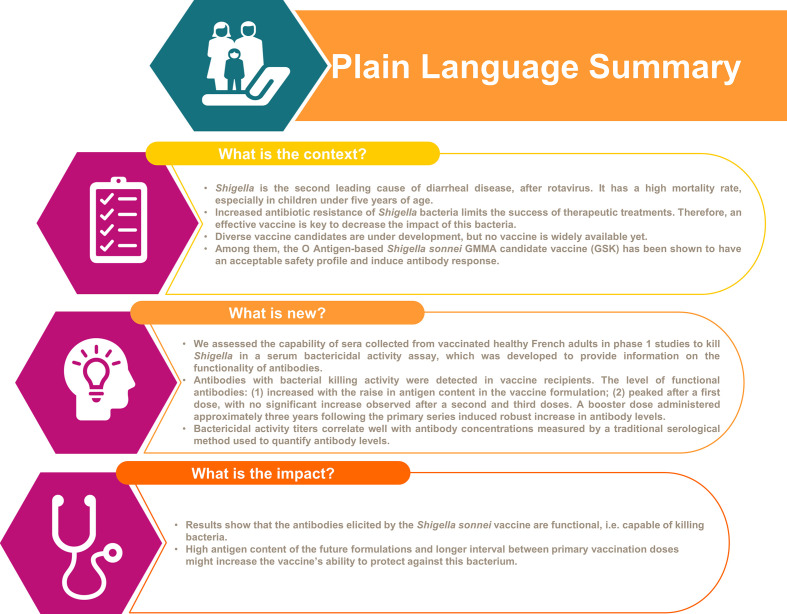
Plain Language Summary.

Here we report bactericidal activity of sera collected from participants of the phase 1 and extension studies using a complement-mediated high-throughput luminescence-based SBA assay developed at the GSK Vaccines Institute for Global Health (39, 40). We also assessed the impact of baseline ELISA antibody levels on SBA titer levels and correlation between ELISA IgG levels and SBA titers. A summary contextualizing the results and potential clinical relevance and impact of the research is provided in the Plain Language Summary (**Figure 1**).

## Material and Methods

### Study Design and Participants

The parent study was a phase 1, observer-blind, randomized, placebo-controlled, dose-escalation study (NCT02017899) conducted in France between February 2014 and March 2015. Detailed study design and eligibility criteria are described in the primary publication (36). Fifty healthy adult volunteers (aged 18–45 years old) were enrolled and received three doses of either one of the following 1790GAHB vaccine formulations or placebo, one month apart: 1790GAHB dose containing 0.06 µg OAg and 1 µg protein (0.06/1 group, 8 participants), 1790GAHB dose containing 0.3 µg OAg and 5 µg protein (0.3/5 group, 9 participants), 1790GAHB dose containing 1.5 µg OAg and 25 µg protein (1.5/25 group, 8 participants), 1790GAHB dose containing 3 µg OAg and 50 µg protein (3/50 group, 8 participants), or 1790GAHB dose containing 6 µg OAg and 100 µg protein (6/100 group, 9 participants). The Placebo group (8 participants) received three doses of Alhydrogel (0.7 mg Al^3+^/dose) in tris-buffered saline.

The extension of the parent trial was a phase 1, open label, non-randomized study (NCT03089879) conducted between March 2017 and August 2017 and designed to evaluate the memory response elicited by a booster dose of 1790GAHB (1.5/25 µg OAg/protein) in adults who had undetectable ELISA antibody levels at baseline and were primed with three vaccine doses containing different amount of OAg (four participants received 0.06/1 µg, one participant 0.3/5 µg, and two participants 3/50 µg OAg/protein dose) 2–3 years earlier in the parent study (Booster group, 7 participants). These participants were compared to vaccine-naïve individuals receiving one 1.5/25 µg 1790GAHB dose (Control group) (38). The Control group consisted of adults who received placebo during the parent study (2 participants) and newly enrolled volunteers (26 participants).

Trials were designed and conducted in accordance with the Good Clinical Practice Guidelines and the Declaration of Helsinki. Written informed consent was obtained before enrollment from each individual. The relevant ethical and regulatory approval was obtained from the respective institutional and national ethics review committees to reuse human serum pool.

### Procedures

Blood samples used in this analysis were collected before the first primary dose (baseline, D1), 28 days after the first (D29), second (D57), and third (D85) primary vaccination, 168 days after the third primary dose (D225) in the parent study, as well as before (pre-booster) and 28 days (D29) and 84 days (D85) after the booster dose in the extension study. Sera were stored at -80°C until further analysis.

The bactericidal activity of antibodies induced by 1790GAHB against *S. sonnei* 53G *virG::cat*, a strain with stabilized major virulence plasmid to ensure stable OAg expression, was evaluated using the high-throughput luminescence-based SBA assay, developed at GSK Vaccines Institute for Global Health (GVGH) and optimized for testing human sera (40). All tested samples were heat inactivated at 56°C for 30 minutes to remove endogenous complement activity. The assay was performed in 96-well round-bottom sterile plates (Corning). Sera were serially diluted, starting from 1:30 dilution, by 2-fold dilution steps up to seven dilution points in Phosphate Buffer Saline at pH 7 (PBS) in the SBA plates (10 µL/well). A reaction mixture of target bacterial cells (20,000 cells/well) and exogenous baby rabbit complement (at 20% final concentration) in PBS (60 µL/well) was added to each well of the SBA plate, mixed and incubated for three hours at 37°C. At the end of the incubation period, the SBA plate was centrifuged at room temperature for 10 minutes at 4000 × *g*. The supernatant was removed, and the remaining live bacterial pellets were resuspended in PBS, transferred to a white round-bottom 96-well plate (Greiner) and mixed 1:1 (*v:v*) with BacTiter-Glo Reagent (Promega). The reaction was incubated for five minutes at room temperature on an orbital shaker, and the luminescence signal was measured by a luminometer (Synergy HT, Biotek). The level of luminescence detected is directly proportional to the number of living bacteria present in the wells, which is inversely proportional to the level of functional antibodies present in the serum (39). The results of the assay are expressed as the IC50, represented by the reciprocal of the serum dilution that results in a 50% reduction of luminescence (correspondent to reciprocal of the serum dilution killing half of the bacteria present in the assay). IC50 were calculated by direct fitting of the raw luminescence data in a 4-parameter logistic regression analysis *versus* the log-transformed serum dilutions. Antibody titers below the lower limit of quantification (LLOQ) (IC50 of 100) were set to half that limit (IC50 of 50) for the purpose of statistical analysis.

### Statistical Analysis

The SBA analysis of sera from the parent study was performed in the modified full analysis set (FAS) including participants who received the study vaccine and whose sera were assayed by ELISA at all five time points. Participants were analyzed as treated and those who did not receive a study vaccine dose but provided a blood sample at a certain time point were excluded from all subsequent analysis. The analysis of sera from the extension study was performed in the FAS comprising participants who received the booster dose and provided at least one evaluable serum sample at a relevant time point.

For each group in both studies, unadjusted SBA geometric mean titers (GMTs) were calculated with their two-sided 95% confidence intervals (CIs) at each time point by exponentiating (base 10) the means and the lower and upper limit of the 95% CIs of the log-transformed titers. Anti-*S. sonnei* LPS serum IgG antibody geometric mean concentrations were calculated with their 95% CIs by exponentiating the mean and 95% CIs of the log-transformed (base 10) ELISA units (EUs). Subjects with missing values were excluded from analyses. Within-group geometric mean ratios and associated 95% CIs were calculated for titers/concentrations at post-vaccination time points *versus* baseline/pre-booster level. Detailed description of anti-*S. sonnei* LPS serum IgG antibody responses were provided in the original publications (36, 38). A post-hoc sub-analysis of SBA GMTs and anti-*S. sonnei* LPS serum IgG antibody concentrations was performed for participants with baseline antibody levels below or above the LLOQ for ELISA. For ELISA testing, the minimum measurable antibody level was determined in each assay and varied from 3.1 to 4.1 EU/mL in the parent study and 5.5 and 7.4 EU/mL in the extension study (36, 38). Antibody concentrations below this level were set at half the minimum measurable level. The number and percentage of participants with a 4-fold increase in SBA titers post-vaccination as compared to pre-vaccination were also computed with 95% CIs. Correlation of SBA GMTs and the anti-*S. sonnei* LPS serum IgG antibody concentrations were assessed by calculating the Pearson correlation coefficients on the log-transformed (base 10) values, for all samples pooled together within studies and by antibodies below and above the ELISA LLOQ at baseline in the parent study.

## Results

### Study Populations

**Figure 2:**
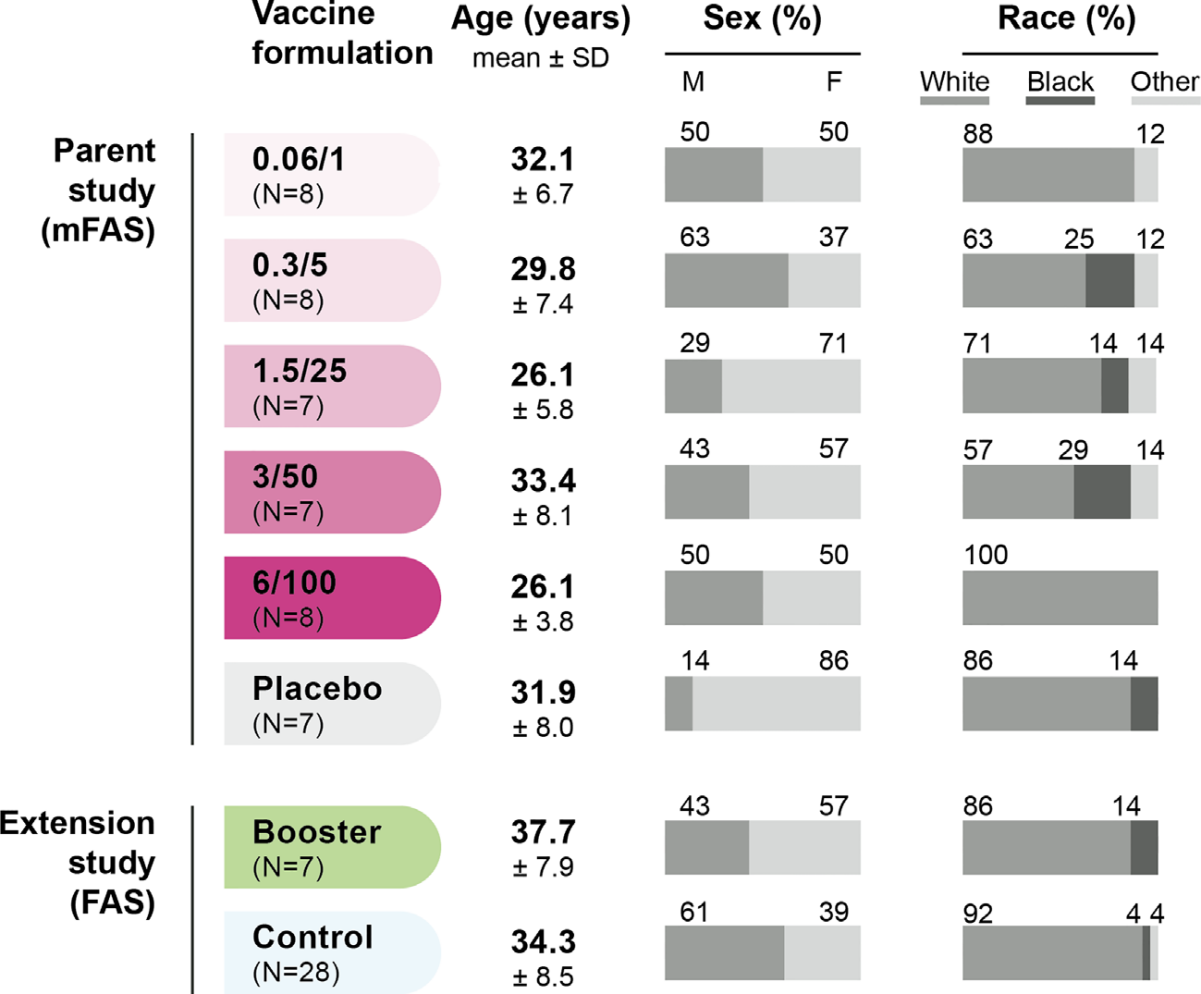
Demographic characteristics of participants. FAS, full analysis set; mFAS, modified FAS; SD, standard deviation; N, number of participants; 0.06/1, group receiving 1790GAHB formulation with 0.06 µg O antigen (OAg) and 1 µg protein; 0.3/5, group receiving 1790GAHB formulation with 0.3 µg OAg and 5 µg protein; 1.5/25, group receiving 1790GAHB formulation with 1.5 µg OAg and 25 µg protein; 3/50, group receiving 1790GAHB formulation with 3 µg OAg and 50 µg protein; 6/100, group receiving 1790GAHB formulation with 6 µg OAg and 100 µg protein; Placebo, group receiving placebo; Booster, group receiving a booster 1790GAHB dose (1.5/25 µg OAg/protein) 2–3 years after primary vaccination; Control, placebo recipients (from the parent study) and vaccine-naïve participants (newly enrolled in the extension study) receiving one dose of 1790GAHB (1.5/25 µg OAg/protein).

Sera from 45 participants of the parent study (38 in 1790GAHB group and seven in Placebo group) were included in the modified FAS of the current analysis. From the extension study, sera from seven participants in 1790GAHB group and 28 participants in Control group were analyzed. Demographic characteristics are presented in **Figure 2**.

### Bactericidal Activity

**Figure 3:**
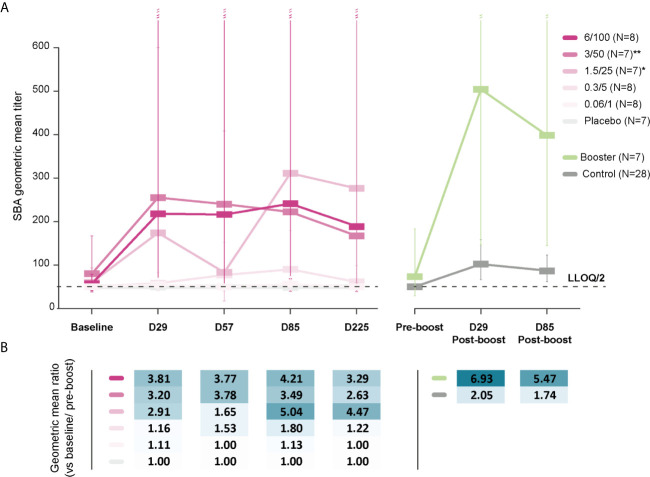
SBA geometric mean titers **(A)** and within-group geometric mean ratios **(B)**. LLOQ, lower limit of quantification; SBA, serum bactericidal activity; Baseline, day of administration of the first dose in the parent study; D29, 28 days post-dose 1; D57, 28 days post-dose 2; D85, 28 days post-dose 3; D225, 6 months post-dose 3; Pre-boost, day of administration of the booster dose (Booster)/vaccine dose (Control) in the extension study; D29 Post-boost; 28 days following booster dose (Booster)/vaccine dose (Control); D85 post-boost, 3 months after the booster dose (Booster)/vaccine dose (Control); N, maximum number of participants with available results; 0.06/1, group receiving 1790GAHB formulation with 0.06 µg O antigen (OAg) and 1 µg protein; 0.3/5, group receiving 1790GAHB formulation with 0.3 µg OAg and 5 µg protein; 1.5/25, group receiving 1790GAHB formulation with 1.5 µg OAg and 25 µg protein; 3/50, group receiving 1790GAHB formulation with 3 µg OAg and 50 µg protein; 6/100, group receiving 1790GAHB formulation with 6 µg OAg and 100 µg protein; Placebo, group receiving placebo; Booster, group receiving a booster 1790GAHB dose (1.5/25 µg OAg/protein) 2–3 years after primary vaccination; Control, placebo recipients (from the parent study) and vaccine-naïve participants (newly enrolled in extension study) receiving one dose of 1790GAHB (1.5/25 µg OAg/protein). *N=4 at D57 and N=6 at D85, D225. **N=6 at D57, D85, D225. Error bars depict 95% confidence intervals.

Baseline SBA GMTs were below the LLOQ in the 0.06/1 and 0.3/5 groups and were 55 (95% CI: 44–70) and 58 (95% CI: 41–83) at D29, respectively. Among participants who received 1790GAHB formulations with high OAg/protein doses, baseline SBA GMTs were 60 (95% CI: 39–93) in the 1.5/25 group, 80 (95% CI: 38–167) in the 3/50 group, and 57 (95% CI: 41–79) in the 6/100 group (**Figure 3A**). At D29, GMTs increased to 174 (95% CI: 51–600) in the 1.5/25 group, 255 (95% CI: 58–1117) in the 3/50 group, and 218 (95% CI: 49–975) in the 6/100 group. At six months following the third dose (D225), GMTs were below the LLOQ in the 0.06/1 group, 61 (95% CI: 38–99) in the 0.3/5 group, 276 (95% CI: 59–1293) in the 1.5/25 group, 167 (95% CI: 39–711) in the 3/50 group, and 189 (95% CI: 69–519) in the 6/100 group. When post-vaccination SBA GMTs were compared with baseline values, fold-increases remained below 1.8 at all time points in the 0.06/1 and 0.3/5 groups (**Figure 3B**). Conversely, SBA GMTs were 2.9-fold [1.5/25 group], 3.2-fold [3/50 group], and 3.8-fold [6/100 group] at D29 and were still 4.5-fold [1.5/25 group], 2.6-fold [3/50 group], and 3.3-fold [6/100 group] above the baseline level at D225 (**Figure 3B**). As expected, no SBA level increase in SBA titers was observed in the Placebo group; antibody levels remained below the LLOQ at all time points. Participants who reached 4-fold increase in bactericidal titers by D29 or D225 were only identified in groups receiving higher OAg/protein doses (**Supplementary Table 1**). Both SBA GMTs and anti-*S. sonnei* LPS serum IgG geometric mean concentrations were highest in groups receiving vaccine doses with higher OAg/protein content, irrespective of baseline antibody levels (**Table 1** and **Supplementary Table 2**).

**Table 1:** Serum bactericidal activity geometric mean titers, anti-*S. sonnei* LPS serum IgG geometric mean concentrations, and within-group geometric mean ratios in participants of the parent study who had baseline antibody levels below the lower limit of quantification for ELISA (modified full analysis set).

Time point	1790GAHB										Placebo	
	N	0.06/1	N	0.3/5	N	1.5/25	N	3/50	N	6/100	N	Value (95% CI)
		Value (95% CI)		Value (95% CI)		Value (95% CI)		Value (95% CI)		Value (95% CI)		
Serum bactericidal activity												
Geometric mean titer												
D1 (baseline)	5	50 (50;50)	4	50 (50;50)	2	50 (N.A.)	4	50 (50;50)	5	50 (50;50)	4	50 (50;50)
D29	5	50 (50;50)	4	50 (50;50)	2	50 (N.A.)	4	205 (13;3181)	5	414 (35;4950)	4	50 (50;50)
D57	5	50 (50;50)	4	50 (50;50)	2	50 (N.A.)	4	225 (12;4172)	5	204 (19;2248)	4	50 (50;50)
D85	5	50 (50;50)	4	78 (19;318)	1	50	4	244 (13;4689)	5	203 (29;1393)	4	50 (50;50)
D225	5	50 (50;50)	4	50 (50;50)	1	50	4	173 (16;1916)	5	154 (36;666)	4	50 (50;50)
Geometric mean ratio												
D29/D1	5	1.00 (1.00;1.00)	4	1.00 (1.00;1.00)	2	1.00 (N.A.)	4	4.10 (0.26;64)	5	8.29 (0.69;99)	4	1.00 (1.00;1.00)
D57/D1	5	1.00 (1.00;1.00)	4	1.00 (1.00;1.00)	2	1.00 (N.A.)	4	4.51 (0.24;83)	5	4.09 (0.37;45)	4	1.00 (1.00;1.00)
D85/D1	5	1.00 (1.00;1.00)	4	1.56 (0.38;6.35)	1	1.00	4	4.89 (0.25;94)	5	4.05 (0.59;28)	4	1.00 (1.00;1.00)
D225/D1	5	1.00 (1.00;1.00)	4	1.00 (1.00;1.00)	1	1.00	4	3.45 (0.31;38)	5	3.09 (0.71;13)	4	1.00 (1.00;1.00)
Anti-*S. sonnei* LPS serum IgG antibodies*												
Geometric mean concentration (EU/mL)												
D1 (baseline)	5	2.57 (2.57;2.57)	4	2.57 (2.57;2.57)	2	2.46 (N.A.)	4	2.17 (1.78;2.65)	5	2.42 (2.13;2.76)	4	2.47 (2.29;2.68)
D29	5	2.57 (2.57;2.57)	4	7.85 (0.69;89)	2	17 (N.A.)	4	118 (8.45;1645)	5	168 (15;1859)	4	2.50 (1.97;3.18)
D57	5	13 (3.65;49)	4	15 (0.77;275)	2	46 (N.A.)	4	75 (3.10;1809)	5	173 (21;1435)	4	2.55 (2.16;3.00)
D85	5	34 (6.00;192)	4	23 (0.66;825)	1	151	4	86 (2.09;3549)	5	137 (20;913)	4	2.41 (1.90;3.06)
D225	5	11 (0.86;135)	4	12 (0.35;387)	1	92	4	61 (1.01;3741)	5	73 (11;500)	4	2.25 (1.10;4.60)
Geometric mean ratio												
D29/D1	5	1.00 (1.00;1.00)	4	3.06 (0.27;35)	2	6.83 (N.A.)	4	54 (4.23;696)	5	70 (5.72;845)	4	1.01 (0.77;1.33)
D57/D1	5	5.23 (1.42;19)	4	5.66 (0.30;107)	2	19 (N.A.)	4	34 (1.57;755)	5	72 (7.94;644)	4	1.03 (0.82;1.29)
D85/D1	5	13.00 (2.34;75)	4	9.11 (0.26;322)	1	61	4	40 (1.04;1516)	5	56 (7.78;409)	4	0.98 (0.72;1.33)
D225/D1	5	4.21 (0.34;53)	4	4.56 (0.14;151)	1	37	4	28 (0.51;1582)	5	30 (4.19;216)	4	0.91 (0.41;1.99)

LPS, lipopolysaccharide; IgG, immunoglobulin G; ELISA, enzyme-linked immunosorbent assay; EU, ELISA unit; D29, 28 days post-dose 1; D57, 28 days post-dose 2; D85, 28 days post-dose 3; D225, 6 months post-dose 3; N, number of participants with available results at a specific time point; 0.06/1, group receiving 1790GAHB formulation with 0.06 µg O antigen (OAg) and 1 µg protein; 0.3/5, group receiving 1790GAHB formulation with 0.3 µg OAg and 5 µg protein; 1.5/25, group receiving 1790GAHB formulation with 1.5 µg OAg and 25 µg protein; 3/50, group receiving 1790GAHB formulation with 3 µg OAg and 50 µg protein; 6/100, group receiving 1790GAHB formulation with 6 µg OAg and 100 µg protein; Placebo, group receiving placebo; CI, confidence interval; N.A., not applicable. CIs were not calculated with N<3. Lower limit of quantification was 100 (IC50) for SBA and 3.1–4.1 EU/mL for ELISA.

*Post-hoc sub-analysis performed on a subset of participants. Anti-S. sonnei LPS serum IgG antibody responses for all study participants has been presented in the primary publication (36).

At pre-booster, SBA GMT was 73 (95% CI: 29–183) in the Booster group and below the LLOQ in the Control group (**Figure 3**). In the Booster group, SBA GMTs increased 6.9-fold to 504 (95% CI: 147–1729) by D29 post-booster and remained as high as 398 (95% CI: 145–1090) at D85 post-booster vaccination. In the Control group, SBA GMTs increased 2.1-fold to 102 (95% CI: 66–158) by D29, subsequently falling to 87 (95% CI: 62–123) by D85 after vaccination. Among participants in the Control group who had pre-vaccination antibody levels above or equal to the LLOQ of the ELISA, SBA GMTs were 131 (95% CI: 72–238) at D29 and 106 (95% CI: 66–172) at D85. By contrast, lower SBA titers (62 [95% CI: 37–105] at D29 and 58 [95% CI: 40–84] at D85) were observed in Control participants with pre-vaccination levels below the LLOQ of the ELISA.

**Figure 4:**
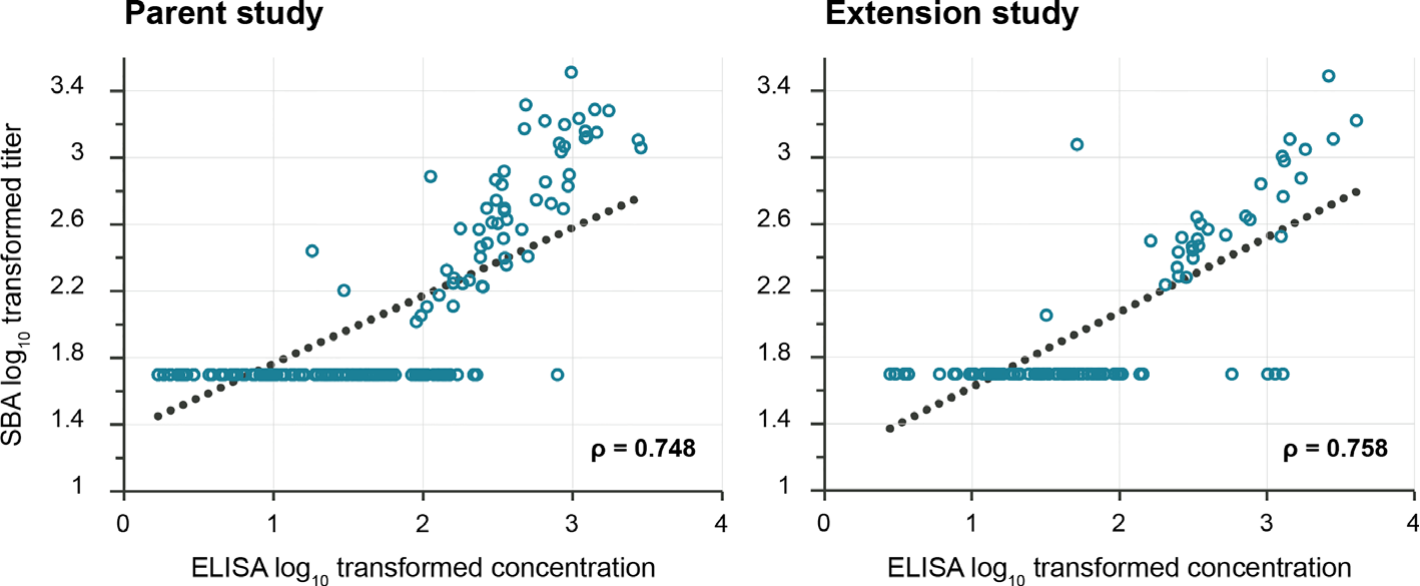
Pearson correlation between anti-*S. sonnei* LPS serum IgG antibody concentrations and SBA titers. LPS, lipopolysaccharide; IgG, immunoglobulin G; SBA, serum bactericidal activity; ELISA, enzyme-linked immunosorbent assay; EU, ELISA unit. The lower limit of quantification was 100 (IC50) for SBA and 3.1–4.1 EU/mL (parent study) and 5.5–7.4 EU/mL (extension study) for ELISA. The p-values were <0.0001 for both coefficients.

Correlation between SBA GMTs and anti-*S. sonnei* LPS IgG antibody concentrations was observed for both studies, the Pearson correlation coefficients being 0.748 in the parent and 0.758 in the extension study (**Figure 4**). In the parent study, similarly high correlation coefficients were calculated for participants with baseline antibody level either below or above the ELISA LLOQ (**Supplementary Figure 1**).

## Discussion

In phase 1 and phase 2 studies, immunogenicity of 1790GAHB has been evaluated in terms of anti-*S. sonnei* LPS serum IgG antibody response (36–38). The current analysis further characterized the immune response elicited by the vaccine and showed the ability of serum antibodies to kill *Shigella* in the SBA assay.

Antibodies with bactericidal activity were detected in sera from 1790GAHB vaccinees and their levels increased with antigen doses. Participants in the 0.06/1 and 0.3/5 groups did not show increase in SBA GMTs at D29 and showed a slight increase at subsequent time points only in the 0.3/5 group. In the 1.5/25, 3/50, and 6/100 groups, however, titers increased at least 2.9-fold by D29 and remained ≥2.6-fold higher by D225 than the SBA titers detected at baseline, which demonstrated the persistence of functional antibodies up to at least six months post-vaccination. The differences among groups clearly indicated an association between the OAg dose in the vaccine and the functional antibody levels elicited by this vaccine. This was also detected in the anti-*S. sonnei* LPS serum IgG antibody concentrations. No bactericidal activity was measured in any participants who had antibody levels below the ELISA LLOQ, demonstrating the specificity of the SBA assay toward this OAg. Bactericidal antibody responses appeared to peak at D29; overall, no further increase in SBA titers was observed after subsequent doses. A booster 1790GAHB dose administered to primed individuals during the extension study induced a robust increase in SBA titers at D29 post-booster, which slightly declined by D85 post-booster, but remained notably higher than post-primary titers. The same trend was observed in the Control group, but at a lower magnitude than in participants in the Booster group. Results obtained in SBA are in line with the observed increase in ELISA concentrations shortly after the booster dose (38). These data suggest that the one-month interval between primary doses is not adequate to further increase immune responses and an interval longer than 28 days between doses might induce higher functional antibody levels. Bactericidal activity titers correlated with anti-*S. sonnei* LPS serum IgG antibody concentrations in both the parent and extension studies as demonstrated by the Pearson correlation coefficients.

In the parent study, a 4-fold increase in functional antibodies, as compared to baseline titers, was detected in participants who received OAg/protein doses of at least 1.5/25 µg and the percentage of participants with a 4-fold increase tended to increase with the OAg dosage. In the extension study, almost half of the vaccinees mounted 4-fold increase post-booster. However, these data should be interpreted with caution. The use of the 4-fold criterion might lead to contradictory results due to its sensitivity to the characteristics and precision of the assay (for continuous assay readouts) or the step size used for serum dilution (for ordinal assay readouts) (41).

Our analysis is affected by the high LLOQ of the luminescence-based assay used to produce this dataset that detracts from the accurate definition of baseline titers. Additionally, this does not allow to obtain precise titers for individuals with low antibody levels, which might consequently result in a less clear dose response and lower correlation between SBA titers and ELISA concentrations. By starting the SBA assay at serum dilution of 1:4 (instead of 1:30), the LLOQ could be reduced to 33 (40), which might allow the analysis of sera with higher sensitivity and better discrimination of participants with pre-existing functional antibodies in future studies. The small sample size can be considered as another potential limitation of this study, which did not allow the assessment of statistical differences.

Other *Shigella* live-attenuated and conjugate vaccine candidates also elicited robust SBA titers in healthy adults following vaccination (21, 24), comparable with titers of individuals from endemic regions who acquire immunity following natural exposure (42). In a recent controlled human infection model, a challenge dose of *S. sonnei* 53G induced LPS-specific serum IgG and IgA antibodies with *S. sonnei*-specific bactericidal activity (43). In contrast, SBA against *S. sonnei* was not detected in adults from endemic settings vaccinated with the live oral *S. sonnei* vaccine (WRSS1), while in children, functional antibody response was significantly higher after three doses of WRSS1 as compared to placebo (44).

SBA reflects the functionality of the antibody response induced and may detect the contribution of multiple immune markers, indicative of a robust response (43). Our high-throughput luminescence-based SBA assay allows the analysis of a high number of samples in a relatively short time (39, 40), thus, can be suitable to perform clinical analysis in future challenge studies or field trials in *Shigella*-endemic regions, and to evaluate the nature of the immune response induced by *Shigella* vaccines.

Functional activities of vaccine-induced *Shigella*-specific antibodies, including both complement-mediated SBA and opsonophagocytosis, may be indicative of clinical protection against shigellosis in humans, as it has been demonstrated for other bacterial pathogens (31). SBA and opsonophagocytic killing antibody (OPKA) titers significantly correlated with reduced illness in patients who received the *Shigella* oral vaccine candidate EcSf2a-2 and were challenged with virulent *S. flexneri* 2a (34). As compared to OPKA, the SBA assay was more sensitive in detecting responses post-challenge and post-vaccination and had more potential to predict the absence of severe disease after challenge (34).

## Conclusion

The results indicated that the serum IgG antibodies elicited by 1790GAHB were functional, with bactericidal activity detectable only in sera from vaccinees, but not from placebo recipients. SBA titers increased with the OAg dosage and sustained responses were detected after the first 1790GAHB dose with OAg/protein content ≥1.5/25 µg. An additional booster dose administered 2–3 years following primary vaccination induced a strong anamnestic response in SBA titers in all primed participants, irrespective of the OAg antigen dose they received during the primary study. SBA titers were found to strongly correlate with anti-*S. sonnei* LPS serum IgG antibodies. The dose response shown in the parent trial, which peaked after the first primary dose, and the strong booster response suggest that high OAg dose and longer interval between primary vaccinations might further increase immunogenicity of this GMMA-based vaccine.

## Data Availability Statement

The original contributions presented in the study are included in the article/**Supplementary Material**. Further inquiries can be directed to the corresponding author.

## Ethics Statement

The studies involving human participants were reviewed and approved by the National Ethic Committee (CPP EST1) assigned according to the pilot phase of the European Union Regulation No. 536/2014 for clinical trial applications in France (for the protocol of the extension trial H03_01E1TP) and CPP Ile-de-France III. Hopital Cochin, Paris, France (for the parent trial H03_01TP). The patients/participants provided their written informed consent to participate in this study.

## Author Contributions

FM, LM, FN, OR, AP, ASS, EM, VC, UN, and OL were involved in the design of the study. FM, FN, OR, EM, and OL performed the study. FM, LM, FN, OR, AP, MA, ASS, EM, VC, OL participated in the collection or generation of study data. FM, LM, FN, OR, and MA contributed with material and reagent tools. FM, LM, FN, OR, AP, ASS, VC, UN, and RR were involved in the analyses and interpretation of the data. LM conceived and coordinated SBA implementation in the overall strategy of the 1790GAHB development. All authors contributed to the article and approved the submitted version.

## Funding

GlaxoSmithKline Biologicals SA funded this analysis and took responsibility for all costs associated with the development of the present manuscript.

## Conflict of Interest

FM, OR, VC, ASS, MA, UN, EM, RR, LM, FN, and AP are employees of the GSK group of companies and RR, LM, and AP hold shares in the GSK group of companies. AS was an employee of the GSK group of companies at the time of the study and holds shares in the GSK group of companies. FM and AS report grant from the Bill and Melinda Gates Foundation during the conduct of the study. LM reports grant from EU FP7 STOPENTERICS during the conduct of the study and from the Bill and Melinda Gates Foundation and Wellcome Trust outside the submitted work. LM and AS are inventors of patents owned by the GSK group of companies and relevant to *Shigella* vaccine. OL’s institution received grant from the GSK group of companies for conducting the study. All authors have no non-financial relationships and activities.

## Abbreviations

OAg, O antigen; LPS, lipopolysaccharide; SBA, serum bactericidal activity; ELISA, enzyme-linked immunosorbent assay; LLOQ, lower limit of quantification; GVGH, GSK Vaccine Institute for Global Health; IgA, immunoglobulin A; IgG, immunoglobulin G; OPKA, opsonophagocytic killing antibody; PBS, phosphate buffer saline; FAS, full analysis set, GMT, geometric mean titer; CI, confidence interval; EU, ELISA unit.
