# Supplementary material for: Antibodies Elicited by the Shigella sonnei GMMA Vaccine in Adults Trigger Complement-Mediated Serum Bactericidal Activity: Results From a Phase 1 Dose Escalation Trial Followed by a Booster Extension
Source: Front Immunol. 2021 May 4;12:671325. doi: 10.3389/fimmu.2021.671325 (PMC8129577; doi:10.3389/fimmu.2021.671325)
Supplement: Supplementary file 1 [file DataSheet_1.docx]

Supplementary Material

# Supplementary Data

**Table S1**. Proportion of participants with 4-fold increase in SBA titers against *S. sonnei* post-vaccination in the parent and extension studies

| **Parent (mFAS)** | **Time point** | **1790GAHB** | | | | | | | | | |  | **Placebo** | |
| --- | --- | --- | --- | --- | --- | --- | --- | --- | --- | --- | --- | --- | --- | --- |
|  |  | **0.06/1** | | **0.3/5** | | **1.5/25** | | **3/50** | | **6/100** | |  |  |  |
|  |  | N | % (95% CI) | N | % (95% CI) | N | % (95% CI) | N | % (95% CI) | N | % (95% CI) |  | N | % (95% CI) |
|  | D29 | 8 | 0 (0.0;36.9) | 8 | 0 (0.0;36.9) | 7 | 14 (0.4;57.9) | 7 | 43 (9.9;81.6) | 8 | 38 (8.5;75.5) |  | 7 | 0 (0.0;41.0) |
|  | D57 | 8 | 0 (0.0;36.9) | 8 | 13 (0.3;52.7) | 4 | 0 (0.0;60.2) | 6 | 33 (4.3;77.7) | 8 | 38 (8.5;75.5) |  | 7 | 0 (0.0;41.0) |
|  | D85 | 8 | 0 (0.0;36.9) | 8 | 0 (0.0;36.9) | 6 | 67 (22.3;95.7) | 6 | 33 (4.3;77.7) | 8 | 38 (8.5;75.5) |  | 7 | 0 (0.0;41.0) |
|  | D225 | 8 | 0 (0.0;36.9) | 8 | 0 (0.0;36.9) | 6 | 50 (11.8;88.2) | 6 | 17 (0.4;64.1) | 8 | 38 (8.5;75.5) |  | 7 | 0 (0.0;41.0) |
| **Extension (FAS)** |  | **Booster** | | | | | | | | | |  | **Control** | |
|  |  | N | % (95% CI) | | | | | | | | |  | N | % (95% CI) |
|  | D29 Post-booster | 7 | 43 (9.9;81.6) | | | | | | | | |  | 28 | 14 (4.0;32.7) |
|  | D85 Post-booster | 7 | 43 (9.9;81.6) | | | | | | | | |  | 28 | 7 (0.9;23.5) |

FAS, full analysis set; mFAS, modified FAS; SBA, serum bactericidal activity; D29, 28 days post-dose 1; D57, 28 days post-dose 2; D85, 28 days post-dose 3; D225, 6 months post-dose 3; D29 Post-booster; 28 days following booster dose (Booster)/ vaccine dose (Control); D85 Post-booster, 3 months after the booster dose (Booster)/ vaccine dose (Control); N, number of participants with available results at a specific time point; 0.06/1, group receiving 1790GAHB formulation with 0.06 µg O antigen (OAg) and 1 µg protein; 0.3/5, group receiving 1790GAHB formulation with 0.3 µg OAg and 5 µg protein; 1.5/25, group receiving 1790GAHB formulation with 1.5 µg OAg and 25 µg protein; 3/50, group receiving 1790GAHB formulation with 3 µg OAg and 50 µg protein; 6/100, group receiving 1790GAHB formulation with 6 µg OAg and 100 µg protein; Placebo, group receiving placebo; Booster, group receiving a booster 1790GAHB dose (1.5/25 µg OAg/protein) 2–3 years after primary vaccination; Control, placebo recipients (from the parent study) and vaccine-naïve participants (newly enrolled in the extension study) receiving one dose of 1790GAHB (1.5/25 µg OAg/protein); CI, confidence interval.

**Table S2**. SBA geometric mean titers, anti-*S. sonnei* LPS serum IgG geometric mean concentrations, and within-group geometric mean ratios in participants of the parent study who had baseline antibody levels above or equal to the lower limit of quantification for ELISA (modified full analysis set)

| **Time point** | **1790GAHB** | | | | | | | | | |  | **Placebo** | | |
| --- | --- | --- | --- | --- | --- | --- | --- | --- | --- | --- | --- | --- | --- | --- |
|  | **0.06/1** | | **0.3/5** | | **1.5/25** | | **3/50** | | **6/100** | |  |  |  |  |
|  | N | Value (95% CI) | N | Value (95% CI) | N | Value (95% CI) | N | Value (95% CI) | N | Value (95% CI) |  | N | Value (95% CI) |  |
| SBA geometric mean titer/ geometric mean ratio | | | | | | | | | | | | | | |
| D1 (baseline) | 3 | 50 (50;50) | 4 | 50 (50;50) | 5 | 64 (32;129) | 3 | 148 (14;1605) | 3 | 72 (15;349) |  | 3 | 50 (50;50) |  |
| D29 | 3 | 66 (20;211) | 4 | 68 (26;178) | 5 | 287 (60;1371) | 3 | 341 (4.7;24845) | 3 | 75 (13;430) |  | 3 | 50 (50;50) |  |
| D57 | 3 | 50 (50;50) | 4 | 118 (23;611) | 2 | 136 (N.A.) | 2 | 273 (N.A.) | 3 | 237 (4.7;11915) |  | 3 | 50 (50;50) |  |
| D85 | 3 | 69 (18;267) | 4 | 104 (27;408) | 5 | 448 (88;2277) | 2 | 184 (N.A.) | 3 | 324 (5.2;20272) |  | 3 | 50 (50;50) |  |
| D225 | 3 | 50 (50;50) | 4 | 75 (21;270) | 5 | 388 (72;2085) | 2 | 157 (N.A.) | 3 | 264 (7.3;9580) |  | 3 | 50 (50;50) |  |
| D29/D1 | 3 | 1.31 (0.41;4.23) | 4 | 1.35 (0.52;3.55) | 5 | 4.47 (0.87;23) | 3 | 2.30 (0.34;16) | 3 | 1.04 (0.88;1.23) |  | 3 | 1.00 (1.00;1.00) |  |
| D57/D1 | 3 | 1.00 (1.00;1.00) | 4 | 2.35 (0.45;12) | 2 | 2.73 (N.A.) | 2 | 2.65 (N.A.) | 3 | 3.29 (0.05;231) |  | 3 | 1.00 (1.00;1.00) |  |
| D85/D1 | 3 | 1.37 (0.35;5.34) | 4 | 2.08 (0.53;8.16) | 5 | 6.96 (1.57;31) | 2 | 1.79 (N.A.) | 3 | 4.49 (0.08;243) |  | 3 | 1.00 (1.00;1.00) |  |
| D225/D1 | 3 | 1.00 (1.00;1.00) | 4 | 1.50 (0.41;5.40) | 5 | 6.04 (1.39;26) | 2 | 1.53 (N.A.) | 3 | 3.66 (0.19;72) |  | 3 | 1.00 (1.00;1.00) |  |
| Anti-*S. sonnei* LPS serum IgG geometric mean concentration (EU/mL)/ geometric mean ratio | | | | | | | | | | | | | |  |
| D1 (baseline) | 3 | 13 (0.90;185) | 5 | 19 (4.07;86) | 6 | 59 (6.10;578) | 3 | 56 (0.25;12574) | 4 | 26 (1.94;338) |  | 4 | 16 (3.75;72) |  |
| D29 | 3 | 27 (1.41;517) | 5 | 54 (9.04;323) | 6 | 286 (43;1877) | 3 | 342 (4.4;26644) | 4 | 144 (15;1373) |  | 4 | 16 (6.80;38) |  |
| D57 | 3 | 31 (1.03;946) | 5 | 95 (27;332) | 6 | 596 (112;3162) | 2 | 243 (N.A.) | 3 | 230 (4.21;12521) |  | 4 | 17 (4.43;64) |  |
| D85 | 3 | 28 (0.54;1417) | 5 | 155 (51;476) | 6 | 590 (116;3009) | 2 | 450 (N.A) | 3 | 204 (3.63;11521) |  | 4 | 25 (4.20;144) |  |
| D225 | 3 | 15 (0.16;1499) | 5 | 117 (50;271) | 6 | 539 (90;3219) | 2 | 318 (N.A.) | 3 | 178 (2.03;15554) |  | 3 | 15 (1.22;182) |  |
| D29/D1 | 3 | 2.09 (0.58;7.47) | 5 | 2.89 (0.84;9.89) | 6 | 4.81 (1.39;17) | 3 | 6.05 (1.80;20) | 4 | 5.62 (1.62;19) |  | 4 | 0.98 (0.52;1.88) |  |
| D57/D1 | 3 | 2.41 (0.62;9.42) | 5 | 5.06 (1.51;17) | 6 | 10 (2.86;35) | 2 | 9.35 (N.A.) | 3 | 5.67 (1.10;29) |  | 4 | 1.03 (0.84;1.26) |  |
| D85/D1 | 3 | 2.15 (0.37;12) | 5 | 8.28 (2.20;31) | 6 | 9.95 (2.54;39) | 2 | 17 (N.A.) | 3 | 5.05 (0.89;29) |  | 4 | 1.50 (0.51;4.43) |  |
| D225/D1 | 3 | 1.19 (0.15;9.69) | 5 | 6.24 (1.57;25) | 6 | 9.08 (2.58;32) | 2 | 12 (N.A.) | 3 | 4.39 (0.98;20) |  | 3 | 1.09 (0.96;1.24) |  |

SBA, serum bactericidal activity; LPS, lipopolysaccharide; IgG, immunoglobulin G; ELISA, enzyme-linked immunosorbent assay; EU, ELISA unit; D29, 28 days post-dose 1; D57, 28 days post-dose 2; D85, 28 days post-dose 3; D225, 6 months post-dose 3; N, number of participants with available results at a specific time point; 0.06/1, group receiving 1790GAHB formulation with 0.06 µg O antigen (OAg) and 1 µg protein; 0.3/5, group receiving 1790GAHB formulation with 0.3 µg OAg and 5 µg protein; 1.5/25, group receiving 1790GAHB formulation with 1.5 µg OAg and 25 µg protein; 3/50, group receiving 1790GAHB formulation with 3 µg OAg and 50 µg protein; 6/100, group receiving 1790GAHB formulation with 6 µg OAg and 100 µg protein; Placebo, group receiving placebo; CI, confidence interval; N.A., not applicable.

Note: CIs were not calculated with N<3. Lower limit of quantification was 100 (IC50) for SBA and 3.1–4.1 EU/mL for ELISA.

**Supplementary Figure 1**. Pearson correlation between anti-*S. sonnei* LPS serum IgG antibody concentrations and SBA titers in participants of the parent study, stratified according to baseline antibody levels below and above the limit of quantification for ELISA (modified full analysis set)


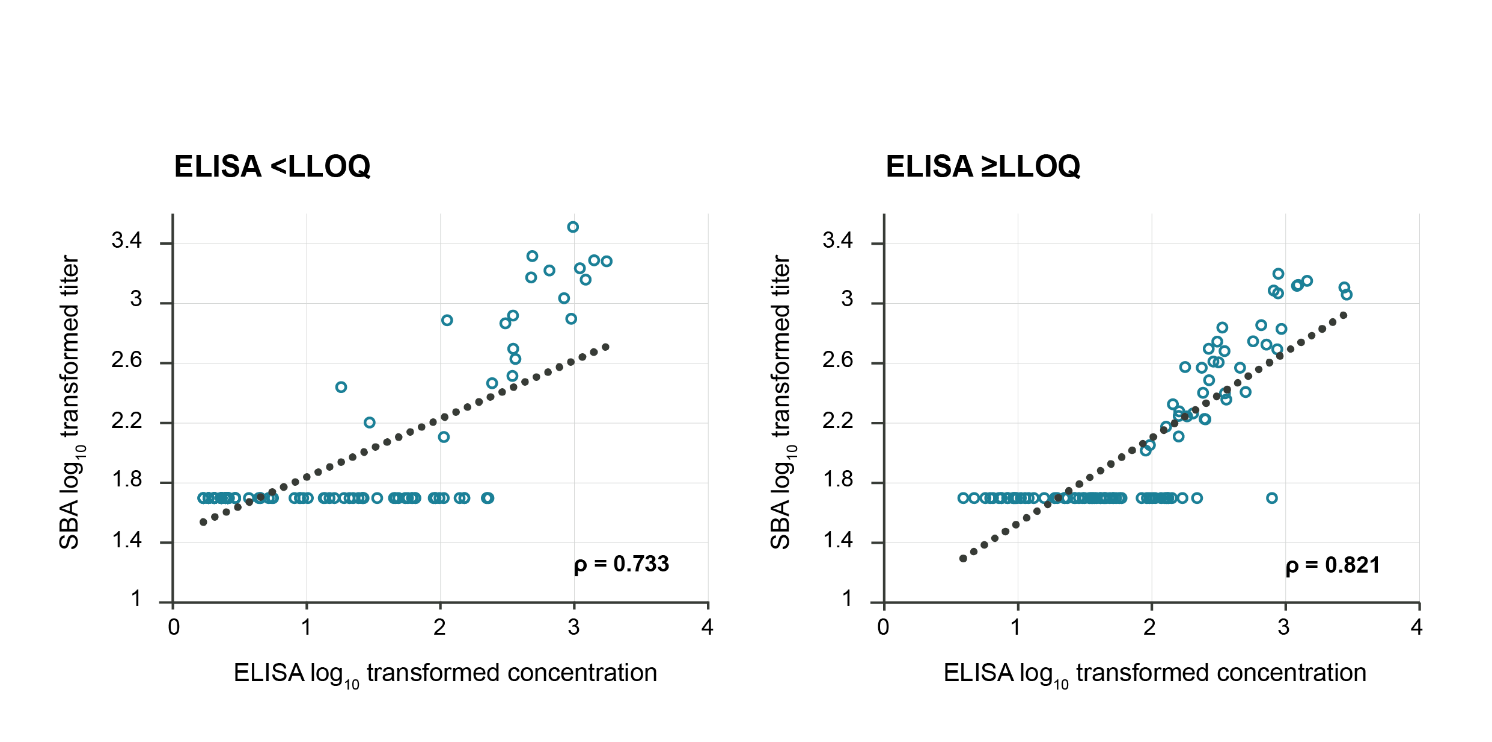


SBA, serum bactericidal activity; LPS, lipopolysaccharide; ELISA, enzyme-linked immunosorbent assay; LLOQ, lower limit of quantification. Note: LLOQ was 100 (IC50) for SBA and 3.1–4.1 EU/mL for ELISA.
